# Items, Selections and Remarks

**Published:** 1869-12

**Authors:** W. W. Miner


### Items, Selections and Remarks.

BY W. W. MINER, A. B.

Dr. G. M. Beard, of New York, in a letter to the *Record*, says that the treatment of nervous diseases by electricity commands in Germany a wider interest than in any other country; and that, during the last ten years, fifty treaties on electro-therapeutics have appeared in Germany alone. In Berlin there are, at present, half a dozen specialists in electro-therapeusis, prominent among whom is Dr. Moritz Meyer, one of the editors of the *Journal of Psychology and Nervous Diseases*, whose excellent treatise Dr. Hammond has just introduced to the profession of America; in the same city, private clinics in this department are held at half-past seven in the morning. In Vienna there are three or four well known authors in this department, chief among whom is Benedict, whose work has attracted considerable attention among the leading neurologists of all countries.

Dr. J A. Ross reports, in the *Lancet*, a case of death, in the Staffordshire Infirmary, from the administration of twenty drops of chloroform. The patient was a miner, fifty years of age, upon whom an operation was to be performed. The chloroform was pure, and was given by means of a cone lint. The pulse stopped suddenly, and the most persevering use of all appliances for restoring animation proved unavailing. The autopsy showed the heart to be rather large and flabby, but otherwise free from disease. The other organs were normal.

The Physicians Mutual Aid Association, of New York, held its annual meeting, Nov. 6th, in the Mott Memorial Rooms, and elected James Anderson, President; Alexander B. Mott, Vice-President; Joseph Monell, Secretary.——More than forty millions of francs have been expended, within thirty years, upon the several civil hospitals in Paris, which contain 19,600 beds. The places of amusement pay a tax of eight per cent. on receipts for support of the hospitals, and a heavy tax is also levied on every piece of ground purchased in the cemeteries.—*Med. Record.* ——Professor H. Knapp, M. D., has opened an Ophthalmic and Aural Institute at No. 46 East 12th Street, New York City.

The names of some of the new anaesthetic agents which are now being experimented with are bichloride of methylene, chloral, bromal, iodal and bromoform. It is probable that none of these will supersede, to any great extent, ether or chloroform, as all of them possess the usual undesirable properties, are sometimes fatal, and would be more or less expensive in price. Chloral is probably valuable as a hypnotic in cases where morphine or the bromides are unsuccessful. Bichloride of methylene has been extensively used in England, and, under the most careful administrators, has produced fatal results in several instances.

E. Michener, M. D., of Taughkenamon, Chester Co., Pa., says, in writing to the *Reporter*, that the use of the "Long Tube in Intestinal Obstructions," according to Dr. Hay, is attended with difficulty; and that he has used, with success, a short anal tube, having a valve within to prevent reflux, and constructed with an external flange at its lower portion, which supports the sphincter ani muscle, and thus enables a large quantity of fluid to be retained. His experience with this tube, which is of his own devising, commenced fifty years since; and he says that, with the tube and any ordinary injecting apparatus, very little difficulty will be experienced in distending the colon and affording speedy relief to the cases requiring its use. The quantity of fluid injected was, in one instance, seven quarts, though the capacity of the colon and rectum, and the quantity of fluid introduced, generally varies from two to six quarts.

Bisulphide of carbon, when purified by washings with pure water, treated with caustic lime and afterwards with copper reduced by hydrogen, is said to have simply a faint etherial odor. M. M. Millon and Commaille, of Paris, have, with this inodorous solvent, extracted the most delicate perfumes, and even from milk have separated the perfume of certain plants which had been eaten.——Dr. Jacobi has presented, to the British Association, a method for the electro-deposition of iron from double salts of that metal. A slight film of copper is first deposited on the matrix to aid the process.

Prof. Joseph Lister, of Glasgow University, whose original experiments in antiseptic surgery are known to our readers, has, at the earnest request of the students of Edinburgh University, been appointed to the chair of surgery, recently vacated in the latter institution by Prof. Syme.——Thomas Graham, M. A., F. R. S., one of the leading English chemists, has recently died. He held the position of Professor of Chemistry in the University College of London for eighteen years, and, for the last fifteen years, that of Master of the Mint. He is well-known by his papers on the Absorption of Gases by Liquids, Dialysis, Colloids and Crystalline substances, the Law of Diffusion of Gases, the Absorption of Hydrogen by the metals, and the suggestion of the name Hydrogenium, as indicating the true nature of that element.

Prof. W. A. Hammond is giving a series of cliniques on Insanity, at the Bellevue Hospital Medical College, on Saturdays at 3½ P. M., to which members of the profession, who desire, are invited to attend.——Dr. John H. Packard, Secretary of the College of Physicians of Philadelphia, has prepared a few simple and important rules for the "Course to be pursued by Bystanders in case of Injury." These are printed as posters, and are furnished at a very low rate to business corporations desiring them.——The efforts to establish a Library for the American Medical Association at Washington, we are happy to say are successful; and the profession are requested to bear in mind that all works deposited there are in excellent keeping, and that the movement will be of great service in the establishing of the medical literature of America.

A method of oxidizing lead and combining it with carbonic acid, being a quick way to make white lead, has been devised. The metal is rolled into thin sheets, and exposed in chambers to the action of steam and to carbonic acid obtained by the oxidation of carbonic oxide of the boiler flues with nitric acid. Another process, is similar to that used in making oxide of zinc. If either of these processes are practicable, it will be a matter of considerable commercial importance.——The anticipations of the mineralogists and miners are directed towards the discovery of tin ore in workable quantities in the United States; but no reliable reports have yet come from any locality, though many unreliable ones have gained confidence and publicity. The latest and largest rumor, an account of which is given in the *American Exchange and Review*, comes from Los Angeles, Cal.

Gray's Anatomy has passed to a fifth edition in England. It has now an Introduction on General Anatomy and Development by Mr. T. Holmes, the well-known author and editor.—*N. Y. Med. Jour.*——The third volume of Prof. Flint's Physiology has just been published.——Theodore Varick, M. D., has been appointed Surgeon General of the State of New Jersey.——Gov. Hoffman has offered a reward of $500 for the detection of the murderer or murderers of Dr. Andrew Mead, of Alleghany.——The bromides have been recommended for the cure of obesity.——Oxalate of cerium is quite highly esteemed for the relief of the vomiting of pregnancy.

The American Presbyterian Mission has a medical missionary department in Canton and vicinity. It has done a great deal of good the past year, treating 50,636 out-patients and 1,058 in-patients, and has performed 1,038 surgical operations, some of them of great importance. A medical class there numbers 12 scholars.——The city of Springfield, Mass., has been more or less infected, the year past, with small pox; and the city fathers are debating as to compelling every inhabitant to be vaccinated immediately.——The Central Ohio Asylum, destroyed by fire some months ago, will be rebuilt, and probably completed, in 1872.—*Med. Record.*

The oldest relic of humanity extant is the skeleton of one of the early Pharaohs, encased in its original burial robes, and wonderfully perfect considering its age, which was deposited about eighteen or twenty months ago in the British Museum, and is justly considered the most valuable of its archæological treasures. The lid of the coffin which contained the royal mummy was inscribed with the name of its occupant, Pharaoh Mikerinus, who succeeded the time of the builder of the Great Pyramid, about ten centuries before Christ. The monarch, whose crumbling bones and leathery integuments are now exciting the wonder gazers in London, reigned in Egypt before Solomon was born, and lived only about eleven centuries or so after Mizraim, the grandson of father Noah, and the first of the Pharaohs had been gathered to his fathers. The tidemark of the deluge would scarcely have been obliterated when this man of the early world lived, moved and had his being.—*Medical Record.*

In the December number of the *Gynaecological Journal*, there is given an account of a very interesting public gathering in Edinburgh, the occasion of which was the presentation, by the Lord Provost, of "a ticket of honorary burgess-ship in the ancient city of Edinburgh" to Sir James Y. Simpson, M D., in recognition of his services to humanity in the alleviation of suffering. This honor has also been previously conferred upon Disraeli, Napier and John Bright. Prof. Simpson's reply to the presentation speech was so excellent and interesting that it hoped that a full account of it may be given elsewhere in the journal.

